# Propranolol inhibits the proliferation, migration and tube formation of hemangioma cells through HIF-1α dependent mechanisms

**DOI:** 10.1590/1414-431X20176138

**Published:** 2017-10-02

**Authors:** Y.Z. Chen, N. Bai, J.H. Bi, X.W. Liu, G.Q. Xu, L.F. Zhang, X.Q. Li, R. Huo

**Affiliations:** 1 Department of Aesthetic, Plastic, and Burn Surgery, Shandong Provincial Hospital Affiliated to Shandong University, Jinan, Shandong, China; 2 Department of Aesthetic, Plastic, and Burn Surgery, Linyi People's Hospital, Linyi, Shandong, China

**Keywords:** Propranolol, Hemangiomas, Hypoxia inducible factor-1α, Knockdown, Regression

## Abstract

The aim of this study was to investigate the mechanism of propranolol on the regression of hemangiomas. Propranolol-treated hemangioma tissues were collected and the expression of hypoxia inducible factor-1α (HIF-1α) was examined. We also established HIF-1α overexpression and knockdown hemangioma cells, and determined the effects of HIF-1α on the hemangioma cells proliferation, apoptosis, migration and tube formation. Significantly increased HIF-1α level was found in the hemangioma tissues compared to that in normal vascular tissues, whereas propranolol treatment decreased the HIF-1α level in hemangioma tissues in a time- and dose-dependent manner. Moreover, propranolol treatment significantly decreased cell proliferation, migration and tube formation as well as promoted cell apoptosis in HIF-1α overexpression and knockdown hemangioma cells. Propranolol suppressed the cells proliferation, migration and tube formation of hemangioma cells through HIF-1α dependent mechanisms. HIF-1α could serve as a novel target in the treatment of hemangiomas.

## Introduction

Infantile hemangioma (IH), one of the most common vascular tumors in infants, is composed of disorganized blood vessels and immature endothelial cells (ECs) (1,2). The prevalence of IH is about 5–10% and it occurs more frequently in female, premature, and low-birth-weight newborns (3,4). Clinicopathologically, IH is a unique category of benign tumors, characterized by abnormal ECs proliferation without a regular vascular architecture during the first year of life, and spontaneous regression of the neovasculature in early childhood. Most IH have a simple course and a general watchful waiting policy is often recommended (5). However, 10% of IHs may grow dramatically, destroy tissue, and impair function, resulting in long-term cutaneous residua, permanent disfigurement, and even danger to life (6). Hence, it is necessary to clarify the relevant mechanism for IH and develop rational therapeutic strategies.

In clinical practice, corticosteroids have been considered the first-line medical management for aggressive IH with local or systemic complications (7). However, the recent advent of propranolol for treatment of severe cases (8) following the unexpected discovery of its effects on hemangioma regression has led to its subsequent popularization of this drug as first-line treatment (9,10). Although some possible explanations for propranolol’s regression have been proposed, there is still no evidence to support this conclusion in hemangiomas. Despite the remarkable effects of propranolol on hemangiomas regression, little is known about its mechanism.

In this study, we collected tissue samples from IH patients and isolated the hemangioma cells for investigating the mechanism of propranolol on the regression of hemangiomas.

## Material and Methods

### Sample collection

From March 2015 to February 2016, 10 IH patients in the present study were recruited for this study and were orally treated with propranolol (Tianjin Lisheng Pharmaceutical Co., Ltd., China). Patients had received no surgery or drug treatments prior to the propranolol treatment. Before and after the propranolol treatment, hemangioma tissues were collected from the patients. The vascular tissues from surgical operations were used as normal control. The study protocol was approved by the Ethics Committee of Shandong Provincial Hospital Affiliated to Shandong University and the written informed consents were provided by family members.

### Isolation of hemangioma cells

According to a previous description, hemangioma cells were isolated from patient tissues (11). Cells were cultured under standard conditions in α-MEM supplemented with 10% FBS, 0.25% fungizone (Sigma-Aldrich, USA) and 1% gentamicin (Sigma-Aldrich). Cells were passaged up to a maximum of five times before use in experiments.

### Reverse transcription-quantitative PCR (RT-qPCR)

Total RNA was isolated using TRIzol Reagent (Invitrogen, USA) according to the manufacturers' instruction. cDNA was synthesized using the first-strand synthesis and qPCR kit (Qiagen, Germany) according to the manufacturer's instructions. RT-qPCR detection was performed using SYBR Green PCR Kit (Qiagen). RT-qPCR conditions were an initial 95°C for 15 min, followed by 40 cycles of 94°C for 15 s, 56°C for 30 s, and 70°C for 30 s. PCR products were subsequently analyzed by a melting curve analysis. Human β-actin mRNA was used as the internal control to normalize the HIF-1α expression and the ^ΔΔ^Ct method was used to analyze the data as described previously (12).

### Western blotting

Cells and tissue samples were lysed in RIPA buffer supplemented with protease inhibitors cocktail, followed by high-speed centrifugation at 16,000 *g* at 4^o^C for 30 min and protein quantification using a bicinchoninic acid assay. The proteins were subjected to sodium dodecyl sulfate-polyacrylamide gel electrophoresis (SDS-PAGE) and the separated proteins were transferred to polyvinylidene difluoride (PVDF, Millipore, USA) membranes. After blocking, the membranes were immunostained with anti-HIF-1α monoclonal primary antibodies (Cell Signaling Technology, USA) overnight, and then the membranes were incubated with appropriate horseradish peroxidase (HRP)-conjugated secondary antibody at room temperature. The protein bands were developed with SuperSignal Ultra Chemiluminescent Substrate (USA). GAPDH (Santa Cruz Biotechnology, USA) was used as the loading control.

### Cell treatment

Hemangioma cells were treated with concentrations of propranolol (0, 5, 50, and 200 μM) for 48 h or treated with 50 μM propranolol for different time courses (24, 48, and 72 h). To overexpress or knockdown HIF-1α protein, hemangioma cells were infected with lentivirus particles overexpressing or with knockdown HIF-1α plasmids (Hanbio, China) for 48 h according to the manufacturer's instructions.

### Cell proliferation

Hemangioma cells (1×10^4^ cells) were seeded onto 96-well plates and cultured in α-MEM supplemented with 10% FBS until they reached 80% confluence. Thereafter, the cells were infected with lentivirus particles overexpressing or with knockdown HIF-1α plasmids with or without propranolol treatment. After 48 h, cellular proliferation was evaluated by CCK-8 kit (Dojindo, Japan) according to the manufacturer's instructions using a microplate reader (Biotek ELX-800, USA) to measure the absorbance.

### Cell apoptosis

Hemangioma cells (1×10^5^ cells) were trypsinized and seeded into 6-well plates in 2 ml of culture medium for attachment. The cells infected with lentivirus particles for 48 h were trypsinized, collected, washed and then stained with annexin V-FITC and propidium iodide (PI; BD Bioscience, USA) for 10 min at 4°C in dark according to a standard protocol. Apoptotic cells were determined by flow cytometry analysis (Becton-Dickinson FACScan, BD Biosciences, USA).

### Transwell migration assay

Hemangioma cells migration assay was determined by transwell system. Briefly, resuspended hemangioma cells (2×10^5^ cells) were seeded onto the upper chamber of the insert (BD Biosciences, 8-µm pore size) coated with matrigel (BD Biosciences), and the lower chamber was filled with α-MEM supplemented with 10% FBS. After maintaining in a cell culture incubator for 12 h the membranes of the upper chamber were stained by crystal violet and 3 random fields (200× magnification) of each membrane were picked for cell counting. All treatments were performed in triplicates.

### Tube formation assay

*In vitro* tube formation assay was performed for assessing the neovascularization capacity. Thawed matrigel (BD Biosciences) was plated onto 96-well plates at 37°C for up to 1 h to form a reconstituted basement membrane. Trypsinized hemangioma cells were harvested and resuspended (1×10^4^ cells/100 µL medium), seeded into matrigel, then incubated at 37°C for 6 h. Tube structures were inspected under an inverted light microscope (200× magnification). The images were quantified using the Image-Pro Plus 6.0 software from Media Cybernetics (USA), as previously described (13). The quantified values are reported as the fold of control.

### Statistical analyses

Statistical analyses were performed using SPSS v18 (USA). Data are reported as means±SD. The Student's *t*-test or one-way analysis of variance was used to determine differences among groups. A P value of less than 0.05 was considered to be significant.

## Results

### Increased expression of HIF-1α in hemangioma tissues

**Figure 1.**
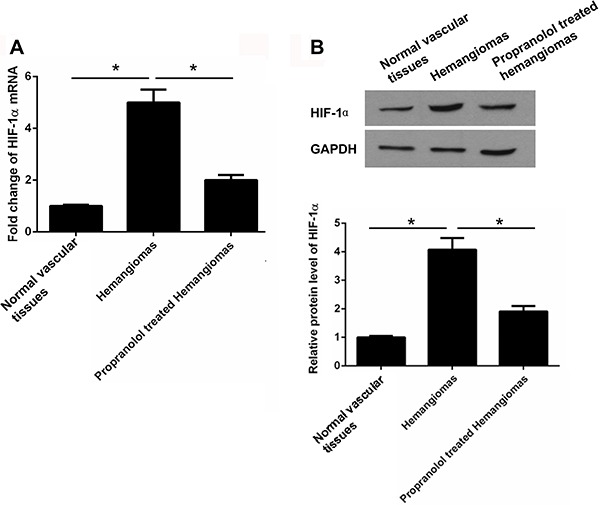
*A*, Expression of hypoxia inducible factor-1α (HIF-1α) mRNA determined by RT-qPCR. Data are reported as fold of control. *B*, Expression of HIF-1α protein level determined by western blotting. Data (lower panel) are reported as the average absorbance of HIF-1α protein of three independent experiments. *P<0.05 (ANOVA).

Significantly increased levels of HIF-1α mRNA and protein were found in hemangioma tissues compared with normal vascular tissues. However, HIF-1α mRNA and protein levels remarkably decreased in the propranolol-treated hemangiomas (Figure 1A and B).

### Propranolol treatment decreased the expression of HIF-1α protein in a dose- and time-dependent manner

**Figure 2.**
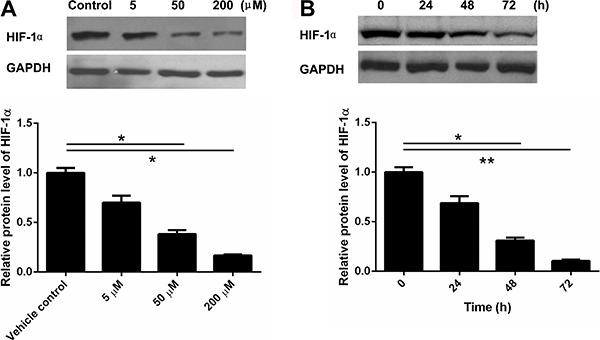
*A*, Hemangioma cells treated with different concentrations of propranolol for 48 h. *B*, Hemangioma cells treated with 50 μM propranolol for 24, 48, and 72 h. The expression of hypoxia inducible factor-1α (HIF-1α) protein level was determined by western blotting. Data are reported as means±SD absorbance of HIF-1α protein of three independent experiments. *P<0.05, **P<0.01 (ANOVA).

The treatment with gradient concentrations of propranolol (0, 5, 50, and 200 μM) showed that it gradually decreased the expression of HIF-1α protein in a dose-dependent manner (Figure 2A). Next, 50 μM propranolol used to treat the hemangioma cells for 24, 48, and 72 h showed that the propranolol gradually decreased the expression of HIF-1α level in a time-dependent manner (Figure 2B).

### Overexpression of HIF-1α abolished the inhibitory effects of propranolol on hemangioma cells proliferation

**Figure 3.**
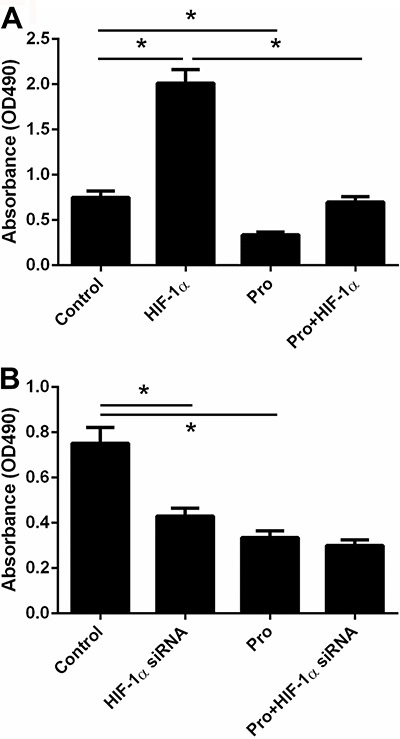
Overexpressed hypoxia inducible factor-1α (HIF-1α) hemangioma cells (*A*) and knockdown hemangioma cells (*B*) were treated or not with 50 μM propranolol (Pro) for 48 h. Cell proliferation was measured by CCK-8 kit assay. Data are reported as means±SD of one representative experiment of at least three experiments with quintuplicates or sextuplicates. *P<0.05 (ANOVA).

To further validate the role of HIF-1α in the propranolol-treated cells, we established HIF-1α overexpressed and knockdown hemangioma cells. The results showed that cell proliferation was significantly inhibited by propranolol treatment of hemangiomas and significantly increased by HIF-1α overexpression, whereas propranolol treatment completely abolished HIF-1α overexpression-induced hemangioma cells proliferation (Figure 3A). Differently, HIF-1α knockdown remarkably inhibited hemangioma cells proliferation (Figure 3B).

### Propranolol treatment promoted hemangioma cell apoptosis

**Figure 4.**
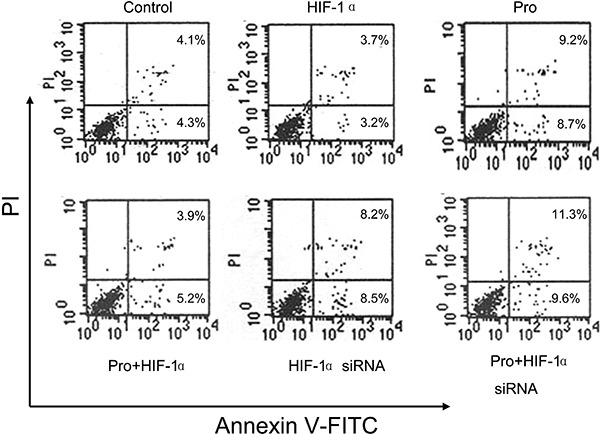
Overexpressed hypoxia inducible factor-1α (HIF-1α) hemangioma cells and knockdown hemangioma cells were treated or not with 50 μM propranolol (Pro) for 48 h. Cell apoptosis was determined by annexin V-FITC. Representative images of cell apoptosis are shown for each group. PI: propidium iodide

As shown in Figure 4, the cell apoptotic percentage in propranolol treatment accounted for 17.9% (9.2+8.7%), almost 2-fold than control group (8.4%, 4.1+4.3%), suggesting that propranolol treatment induced hemangioma cell apoptosis. On the other hand, the cell apoptotic percentage accounted for 9.1% (3.9+5.2%) in HIF-1α overexpression group and 16.7% (8.2+8.5%) in HIF-1α knockdown group, suggesting that HIF-1α overexpression abrogated propranolol-induced hemangioma cells apoptosis.

### Overexpression of HIF-1α abrogated the inhibitory effects of propranolol on hemangioma cells migration

**Figure 5.**
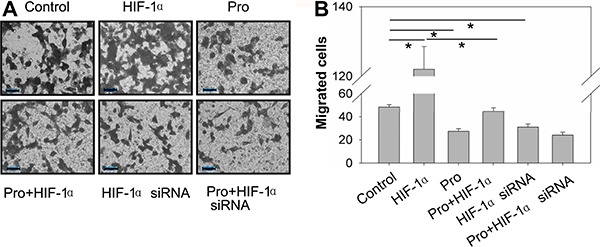
Overexpressed hypoxia inducible factor-1α (HIF-1α) hemangioma cells and knockdown hemangioma cells were treated or not with 50 μM propranolol (Pro) for 48 h. Cell migration was measured by transwell system. *A*, Representative images of cells migration (200× magnification). *B*, Quantified values of migrated cells in each group. Data are reported as means±SD of 3 random fields. All treatments were performed in triplicate. *P<0.05 (ANOVA).

The results showed that propranolol treatment decreased the number of migrated cells. Contrarily, HIF-1α overexpression significantly increased the cell migration and HIF-1α knockdown significantly decreased cell migration. Propranolol treatment completely abrogated the HIF-1α overexpression-increase in migrated cells (Figure 5A and B).

### Overexpression of HIF-1α abrogated the inhibitory effects of propranolol on tube formation in hemangioma cells

**Figure 6.**
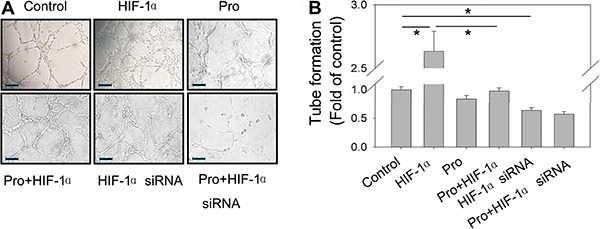
Overexpressed hypoxia inducible factor-1α (HIF-1α) hemangioma cells and knockdown hemangioma cells treated or not with 50 μM propranolol (Pro) for 48 h. *A*, Representative images of tube formation (200× magnification). *B*, Relative quantified values of numbers of tubes in each group. Data are reported as means±SD of 3 random fields. All treatments were performed in triplicate. *P<0.05 (ANOVA).

The tube formation test results showed that propranolol treatment slightly decreased the level of tube formation. HIF-1α overexpression, on the other hand, significantly increased the level of tube formation and HIF-1α knockdown decreased the level of tube formation. Propranolol treatment completely abrogated the HIF-1α overexpression-increase in tube formation (Figure 6A and B).

## Discussion

In the present study, we demonstrated that significantly increased HIF-1α levels were found in hemangioma tissues compared to normal vascular tissues, and its levels were decreased by propranolol treatment. The *in vitro* study demonstrated that propranolol treatment decreased the expression of HIF-1α protein in a dose- and time-dependent manner. Moreover, propranolol treatment significantly decreased hemangioma cell proliferation, migration and tube formation in HIF-1α-overexpressed and knockdown cells.

Traditionally, corticosteroids as well as other drugs such as interferon α and vincristine have been considered as first-line therapeutic approaches for the treatment of IH. However, multiple and serious side-effects that are very painful accompany these drugs. Recent evidence shows that propranolol effectively regressed IH in a patient with obstructive hypertrophic cardiomyopathy. Further evidence has shown that the use of propranolol in the treatment of IH can achieve satisfactory outcomes and no serious side-effects. Furthermore, propranolol may be effective in IH patients who are allergic to corticosteroids and interferon or in severe IH. It has been suggested that propranolol exerts its effects as a non-selective β-adrenergic receptor (AR) blocker that inhibits cell growth and induces cell apoptosis of the endothelial cells (14). Our results confirmed the cytotoxic effect of propranolol with an annexin V-FITC apoptosis assay, which demonstrated that high concentrations of propranolol resulted in increased apoptosis. However, the precise molecular mechanism of its action remains poorly understood.

Studies have shown that a causal relationship are observed between the expressions of β-AR and HIF-1α in pancreatic, breast and prostate cancer cells (15,16). Accumulation of HIF-1α was found to correlate with β-adrenergic agonists treatment, whereas norepinephrine-induced expressions of HIF-1α and vascular endothelial growth factor (VEGF) are blocked by propranolol treatment. Besides HIF-1α, there are numbers of other VEGF regulators. Several other non-canonical pathways are not dependent on hypoxia and HIF-1α also regulate VEGF and angiogenesis (17,18). However, several studies have demonstrated that children with proliferative hemangiomas showed upregulation and stabilization of HIF-1α compared to normal controls (19–21), suggesting that these lesions may be intrinsically hypoxic, resulting in hemangioma endothelial cell proliferation through VEGF-mediated downstream signaling. This intrinsic difference in HIF-1α expression between hemangiomas and normal tissues could result in predominance of the canonical HIF-1α pathway in VEGF signaling. A reliance on HIF-1α signaling and dependence on VEGF (22) for survival could explain the specific response of hemangiomas rather than other tissues treated with propranolol.

In conclusion, significantly increased HIF-1α levels were found in the hemangioma tissues. Propranolol treatment inhibited cell proliferation, migration, tube formation and promoted apoptosis in the hemangioma cells, suggesting that HIF-1α could serve as a therapeutic target in the treatment of IH.
